# *N*,*N*′-(Pyridine-2,6-di­yl)dibenzamide

**DOI:** 10.1107/S1600536812050167

**Published:** 2012-12-19

**Authors:** Parisa Mahboubi Anarjan, Nader Noshiranzadeh, Rahman Bikas, Magdalena Woińska, Krzysztof Wozniak

**Affiliations:** a Sama Technical and Vocational Training College, Islamic Azad University, Mamaghan Branch, Mamaghan, Iran; b Department of Chemistry, University of Zanjan, 45195-313 Zanjan, Iran; c Chemistry Department, The University of Warsaw, Pasteura 1, 02093 Warszawa, Poland

## Abstract

The mol­ecule of the title compound, C_19_H_15_N_3_O_2_, is completed by the application of crystallographic twofold symmetry, with the pyridine N atom lying on the rotation axis. The mol­ecular structure is approximately planar, the dihedral angle between the mean planes of the pyridine and benzene rings being 7.53 (11)°. In the crystal, N—H⋯O hydrogen bonds link the mol­ecules into a two-dimensional array perpendicular to the *c* axis.

## Related literature

For metal complexes of carboxamide ligands, see: Adolph *et al.* (2012 ▶); Amiri *et al.* (2009 ▶).
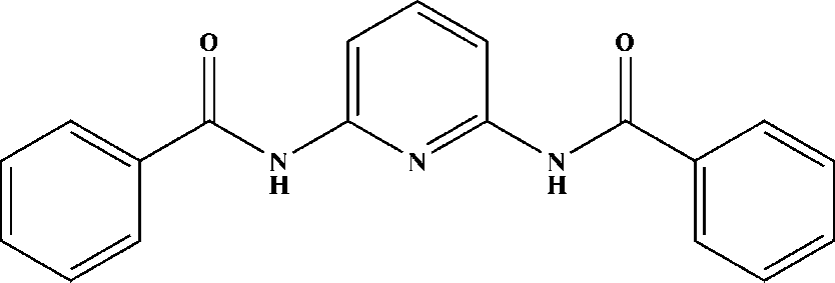


## Experimental

### 

#### Crystal data

C_19_H_15_N_3_O_2_
*M* *_r_* = 317.34
Tetragonal, 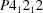

*a* = 5.0314 (1) Å
*c* = 58.701 (3) Å
*V* = 1486.02 (8) Å^3^
*Z* = 4
Mo *K*α radiation
μ = 0.10 mm^−1^
*T* = 100 K
0.21 × 0.09 × 0.02 mm

#### Data collection

Oxford Diffraction Xcalibur Opal diffractometer
Absorption correction: multi-scan (*CrysAlis RED*; Oxford Diffraction, 2010 ▶) *T*_min_ = 0.986, *T*_max_ = 1.000
1298 measured reflections
16463 independent reflections
1168 reflections with *I* > 2σ(*I*)
*R*_int_ = 0.065

#### Refinement

*R*[*F* ^2^ > 2σ(*F* ^2^)] = 0.044
*wR*(*F* ^2^) = 0.090
*S* = 1.23
1298 reflections
112 parameters
H-atom parameters constrained
Δρ_max_ = 0.18 e Å^−3^
Δρ_min_ = −0.16 e Å^−3^

### 

Data collection: *CrysAlis CCD* (Oxford Diffraction, 2010 ▶); cell refinement: *CrysAlis CCD*; data reduction: *CrysAlis RED* (Oxford Diffraction, 2010 ▶); program(s) used to solve structure: *SHELXS97* (Sheldrick, 2008 ▶); program(s) used to refine structure: *SHELXL97* (Sheldrick, 2008 ▶); molecular graphics: *WinGX* (Farrugia, 2012 ▶) and *DIAMOND* (Brandenburg & Putz, 2006 ▶); software used to prepare material for publication: *publCIF* (Westrip, 2010 ▶).

## Supplementary Material

Crystal structure: contains datablock(s) I, global. DOI: 10.1107/S1600536812050167/tk5180sup1.cif

Structure factors: contains datablock(s) I. DOI: 10.1107/S1600536812050167/tk5180Isup2.hkl

Supplementary material file. DOI: 10.1107/S1600536812050167/tk5180Isup3.cml

Additional supplementary materials:  crystallographic information; 3D view; checkCIF report

## Figures and Tables

**Table 1:** Hydrogen-bond geometry (Å, °)

*D*—H⋯*A*	*D*—H	H⋯*A*	*D*⋯*A*	*D*—H⋯*A*
N2—H2⋯O1^i^	0.86	2.25	3.030 (2)	151

Symmetry code: (i) 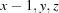.

**Fig. 1.**
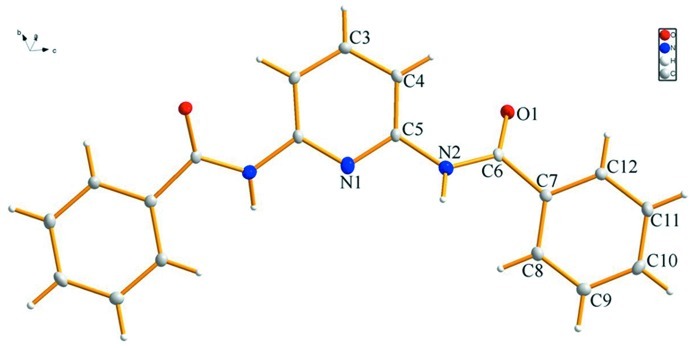
Molecular structure of the title compound showing displacement ellipsoids at the 50% probability level. The molecule has twofold symmetry and the unlabelled atoms are related by the symmetry operation y, x, -z.

**Fig. 2.**
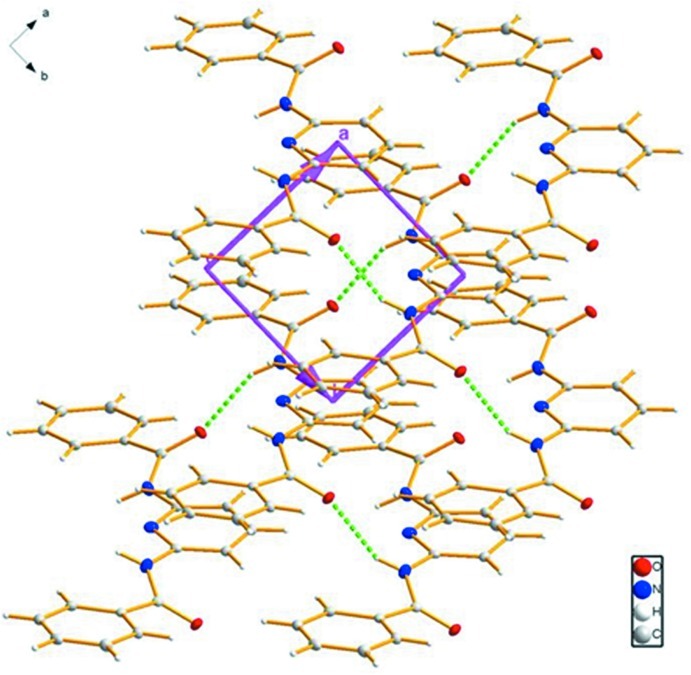
Hydrogen bonding in the title compound leading to supramolecular layers in the ab plane. The green dashed lines indicate N—H···O hydrogen bonds.

## supplementary crystallographic information

### Comment

Carboxamides are compounds which are prepared by the reaction of amines and acylhalides. They are important *N*,*O*-donor ligands and have widespread applications in fields such as in coordination chemistry (Adolph *et al.* 2012 & Amiri *et al.* 2009). As biologically active compounds, carboxamides find application in the treatment of diseases such as cancer, rheumatic disorders and inhibitors of calpain (calcium dependant cysteine proteases).

The molecular structure of the title compound is shown in Fig. 1. The molecule is approximately planar with the dihedral angle between the mean planes of the pyridine and benzene rings being 7.53 (11)°. Intermolecular N—H···O hydrogen bonds, with the carbonyl-O atoms acting as acceptors, link molecules into a two-dimensional array perpendicular to the *c* axis as illustrated in Fig. 2.

### Experimental

All reagents were commercially available and used as received. To a magnetically stirred solution of 2,6-diaminopyridine (0.109 g, 1 mmol) and triethylamine (0.277 ml, 2 mmol) in dichloromethane (5 ml) was added drop-wise a mixture of benzoyl chloride (0.232 ml, 2 mmol) in dichloromethane (2 ml) at -10 °C over 15 min. The mixture was allowed to warm to room temperature and stirred for 48 h at room temperature. The solvent was removed under reduced pressure and the residue was purified by flash column chromatography (silica gel; petroleum ether-ethyl acetate) to give the title compound as a yellow powder. Crystals of the title compound were obtained from its methanol solution by slow solvent evaporation. Yield: 85%. Melting point: 407–408 K. Selected IR (KBr, cm^-1^): 3245 (N—H), 3061 (C—H), 1653 (C═O_amide_), 1584 (C═N), 1461 (C═C).

### Refinement

The hydrogen atom of the N—H group was positioned geometrically and refined as a riding atoms with N—H = 0.86 Å, and with *U*_is__o_(H) = 1.2*U*_eq_(N). The C—H hydrogen atoms were positioned geometrically and refined as riding atoms with C—H = 0.93 Å, and with *U*_iso_(H) = 1.2*U*_eq_(C).

### Crystal data

**Table:**

C_19_H_15_N_3_O_2_	*D*_x_ = 1.418 Mg m^−^^3^
*M**_r_* = 317.34	Mo *K*α radiation, λ = 0.71073 Å
Tetragonal, *P*4_1_2_1_2	Cell parameters from 3266 reflections
Hall symbol: P 4abw 2nw	θ = 1.7–28.5°
*a* = 5.0314 (1) Å	µ = 0.10 mm^−^^1^
*c* = 58.701 (3) Å	*T* = 100 K
*V* = 1486.02 (8) Å^3^	Block, yellow
*Z* = 4	0.21 × 0.09 × 0.02 mm
*F*(000) = 664	

### Data collection

**Table:**

Oxford Diffraction Xcalibur Opal diffractometer	1298 independent reflections
Radiation source: fine-focus sealed tube	1168 reflections with *I* > 2σ(*I*)
Graphite monochromator	*R*_int_ = 0.065
Detector resolution: 8.4441 pixels mm^-1^	θ_max_ = 24.9°, θ_min_ = 2.8°
ω scan	*h* = 0→5
Absorption correction: multi-scan (*CrysAlis RED*; Oxford Diffraction, 2010)	*k* = 0→4
*T*_min_ = 0.986, *T*_max_ = 1.000	*l* = −64→68
16463 measured reflections	

### Refinement

**Table:**

Refinement on *F*^2^	Secondary atom site location: difference Fourier map
Least-squares matrix: full	Hydrogen site location: inferred from neighbouring sites
*R*[*F*^2^ > 2σ(*F*^2^)] = 0.044	H-atom parameters constrained
*wR*(*F*^2^) = 0.090	*w* = 1/[σ^2^(*F*_o_^2^) + (0.*P*)^2^ + 0.9748*P*] where *P* = (*F*_o_^2^ + 2*F*_c_^2^)/3
*S* = 1.23	(Δ/σ)_max_ < 0.001
1298 reflections	Δρ_max_ = 0.18 e Å^−^^3^
112 parameters	Δρ_min_ = −0.16 e Å^−^^3^
0 restraints	Extinction correction: *SHELXL97* (Sheldrick, 2008), Fc^*^=kFc[1+0.001xFc^2^λ^3^/sin(2θ)]^-1/4^
Primary atom site location: structure-invariant direct methods	Extinction coefficient: 0.0091 (16)

### Special details

**Table:**

Geometry. All e.s.d.'s (except the e.s.d. in the dihedral angle between two l.s. planes) are estimated using the full covariance matrix. The cell e.s.d.'s are taken into account individually in the estimation of e.s.d.'s in distances, angles and torsion angles; correlations between e.s.d.'s in cell parameters are only used when they are defined by crystal symmetry. An approximate (isotropic) treatment of cell e.s.d.'s is used for estimating e.s.d.'s involving l.s. planes.
Refinement. Refinement of *F*^2^ against ALL reflections. The weighted *R*-factor *wR* and goodness of fit *S* are based on *F*^2^, conventional *R*-factors *R* are based on *F*, with *F* set to zero for negative *F*^2^. The threshold expression of *F*^2^ > σ(*F*^2^) is used only for calculating *R*-factors(gt) *etc*. and is not relevant to the choice of reflections for refinement. *R*-factors based on *F*^2^ are statistically about twice as large as those based on *F*, and *R*- factors based on ALL data will be even larger.

### Fractional atomic coordinates and isotropic or equivalent isotropic displacement parameters (Å^2^)

**Table:**

	*x*	*y*	*z*	*U*_iso_*/*U*_eq_	
O1	1.3618 (3)	0.6195 (3)	0.04884 (3)	0.0204 (4)	
N1	0.8073 (4)	0.8073 (4)	0.0000	0.0135 (6)	
N2	0.9407 (4)	0.6428 (4)	0.03476 (3)	0.0143 (5)	
H2	0.7819	0.5819	0.0362	0.017*	
C7	1.0307 (5)	0.3655 (5)	0.06778 (4)	0.0144 (5)	
C5	0.9853 (5)	0.8265 (5)	0.01690 (3)	0.0138 (5)	
C8	0.8160 (5)	0.1943 (5)	0.06481 (4)	0.0171 (5)	
H8	0.7180	0.1997	0.0514	0.020*	
C3	1.1969 (5)	1.1969 (5)	0.0000	0.0159 (7)	
H3	1.3276	1.3276	0.0000	0.019*	
C6	1.1258 (5)	0.5538 (5)	0.04983 (4)	0.0143 (5)	
C12	1.1738 (5)	0.3566 (5)	0.08806 (4)	0.0168 (5)	
H12	1.3187	0.4690	0.0901	0.020*	
C4	1.1857 (5)	1.0149 (5)	0.01758 (4)	0.0154 (5)	
H4	1.3082	1.0188	0.0294	0.018*	
C11	1.1026 (5)	0.1821 (5)	0.10523 (4)	0.0210 (6)	
H11	1.1967	0.1802	0.1189	0.025*	
C9	0.7480 (5)	0.0157 (5)	0.08184 (4)	0.0186 (6)	
H9	0.6066	−0.1007	0.0797	0.022*	
C10	0.8902 (5)	0.0100 (5)	0.10204 (4)	0.0200 (6)	
H10	0.8434	−0.1091	0.1135	0.024*	

### Atomic displacement parameters (Å^2^)

**Table:**

	*U*^11^	*U*^22^	*U*^33^	*U*^12^	*U*^13^	*U*^23^
O1	0.0118 (9)	0.0265 (10)	0.0231 (9)	−0.0002 (7)	−0.0012 (7)	0.0046 (8)
N1	0.0144 (9)	0.0144 (9)	0.0118 (13)	0.0035 (12)	0.0000 (8)	0.0000 (8)
N2	0.0115 (11)	0.0178 (11)	0.0135 (9)	−0.0022 (9)	−0.0010 (8)	0.0016 (8)
C7	0.0149 (12)	0.0137 (12)	0.0147 (12)	0.0023 (10)	0.0005 (10)	−0.0025 (10)
C5	0.0164 (12)	0.0140 (13)	0.0110 (11)	0.0045 (10)	0.0008 (10)	−0.0007 (9)
C8	0.0165 (13)	0.0180 (13)	0.0167 (12)	0.0031 (11)	−0.0013 (10)	−0.0004 (10)
C3	0.0157 (11)	0.0157 (11)	0.0162 (17)	−0.0016 (14)	0.0011 (10)	−0.0011 (10)
C6	0.0175 (13)	0.0160 (13)	0.0094 (11)	0.0022 (10)	0.0003 (10)	−0.0033 (9)
C12	0.0197 (13)	0.0164 (13)	0.0143 (11)	−0.0016 (11)	−0.0006 (10)	−0.0013 (10)
C4	0.0144 (13)	0.0181 (13)	0.0136 (11)	0.0018 (10)	−0.0018 (10)	−0.0024 (10)
C11	0.0262 (14)	0.0210 (14)	0.0158 (12)	0.0011 (11)	−0.0039 (10)	0.0007 (11)
C9	0.0175 (13)	0.0173 (13)	0.0211 (12)	0.0003 (11)	0.0018 (11)	−0.0015 (10)
C10	0.0254 (14)	0.0162 (13)	0.0184 (12)	0.0024 (11)	0.0062 (11)	0.0038 (11)

### Geometric parameters (Å, º)

**Table:**

O1—C6	1.234 (3)	C3—C4^i^	1.381 (3)
N1—C5	1.340 (3)	C3—C4	1.381 (3)
N1—C5^i^	1.340 (3)	C3—H3	0.9300
N2—C6	1.360 (3)	C12—C11	1.384 (3)
N2—C5	1.415 (3)	C12—H12	0.9300
N2—H2	0.8600	C4—H4	0.9300
C7—C12	1.392 (3)	C11—C10	1.388 (3)
C7—C8	1.393 (3)	C11—H11	0.9300
C7—C6	1.496 (3)	C9—C10	1.385 (3)
C5—C4	1.385 (3)	C9—H9	0.9300
C8—C9	1.387 (3)	C10—H10	0.9300
C8—H8	0.9300		
			
C5—N1—C5^i^	116.9 (3)	O1—C6—C7	120.7 (2)
C6—N2—C5	126.0 (2)	N2—C6—C7	116.6 (2)
C6—N2—H2	117.0	C11—C12—C7	120.6 (2)
C5—N2—H2	117.0	C11—C12—H12	119.7
C12—C7—C8	119.2 (2)	C7—C12—H12	119.7
C12—C7—C6	117.2 (2)	C3—C4—C5	117.5 (2)
C8—C7—C6	123.5 (2)	C3—C4—H4	121.2
N1—C5—C4	123.9 (2)	C5—C4—H4	121.2
N1—C5—N2	113.3 (2)	C12—C11—C10	119.8 (2)
C4—C5—N2	122.8 (2)	C12—C11—H11	120.1
C9—C8—C7	120.1 (2)	C10—C11—H11	120.1
C9—C8—H8	119.9	C10—C9—C8	120.2 (2)
C7—C8—H8	119.9	C10—C9—H9	119.9
C4^i^—C3—C4	120.3 (3)	C8—C9—H9	119.9
C4^i^—C3—H3	119.9	C9—C10—C11	120.0 (2)
C4—C3—H3	119.9	C9—C10—H10	120.0
O1—C6—N2	122.7 (2)	C11—C10—H10	120.0
			
C5^i^—N1—C5—C4	−0.84 (17)	C8—C7—C6—N2	28.4 (3)
C5^i^—N1—C5—N2	176.1 (2)	C8—C7—C12—C11	−0.7 (4)
C6—N2—C5—N1	156.80 (19)	C6—C7—C12—C11	−178.2 (2)
C6—N2—C5—C4	−26.3 (3)	C4^i^—C3—C4—C5	−0.75 (15)
C12—C7—C8—C9	−0.6 (3)	N1—C5—C4—C3	1.6 (3)
C6—C7—C8—C9	176.7 (2)	N2—C5—C4—C3	−174.99 (17)
C5—N2—C6—O1	−2.8 (4)	C7—C12—C11—C10	1.5 (4)
C5—N2—C6—C7	178.16 (19)	C7—C8—C9—C10	1.2 (3)
C12—C7—C6—O1	26.8 (3)	C8—C9—C10—C11	−0.4 (4)
C8—C7—C6—O1	−150.6 (2)	C12—C11—C10—C9	−0.9 (4)
C12—C7—C6—N2	−154.2 (2)		

Symmetry code: (i) *y*, *x*, −*z*.

### Hydrogen-bond geometry (Å, º)

**Table:**

*D*—H···*A*	*D*—H	H···*A*	*D*···*A*	*D*—H···*A*
N2—H2···O1^ii^	0.86	2.25	3.030 (2)	151

Symmetry code: (ii) *x*−1, *y*, *z*.
